# Comparative Biomechanical Evaluation of Bicortical Screw Versus Plate Fixation in Jones Fractures of the Fifth Metatarsal Using 3D-Printed Models

**DOI:** 10.3390/jcm14207449

**Published:** 2025-10-21

**Authors:** Robert Daniel Dobrotă, Mark Pogărășteanu, Dumitru Ferechide, Ioana-Codruța Lebada, Marius Moga

**Affiliations:** 1 Faculty of Medicine, Carol Davila University of Medicine and Pharmacy, 37 Dionisie Lupu Street, 020021 Bucharest, Romaniamarius.moga@umfcd.ro (M.M.); 2 Dr. Carol Davila Central University Emergency Military Hospital, 010825 Bucharest, Romania; 3 Faculty of Medicine, Lucian Blaga University of Sibiu, 550024 Sibiu, Romania

**Keywords:** 3D printing, bicortical screw, fifth metatarsal fracture, fixation stability, gait biomechanics, Jones fracture, mechanical testing, plate, surgical decision-making

## Abstract

**Background:** Jones fractures of the 5th metatarsal are frequently associated with nonunion due to limited vascularization and repetitive mechanical stress. The aim of the study was to compare the biomechanical performance of T-plate and bicortical screw fixation using standardized 3D models. **Methods:** Three-dimensional models of the 5th metatarsal were generated from CT images and printed using PolyJet technology (Stratasys J5 DentaJet) using a rigid-elastic composite with properties similar to cortical and cancellous bone. Jones fractures were fixed with either a locked T-plate or a bicortical screw. The samples were tested under axial and oblique static loads (α = 0°, 90°, 180°) and for three values of interfragmentary distance (d = 0.1–1 mm), in a 3 × 2 factorial design. **Results:** The T-plate fixation recorded a maximum yield force (Fmax) of 149.78 ± 8.53 N (138–161 N), significantly higher compared to the bicortical screw −98.56 ± 2.58 N (96–101 N), (*p* < 0.05). The ductility index was higher for the plate, indicating a progressive transition to yield. The α and d factors significantly influenced the mechanical behavior, with the polynomial model explaining over 95% of the total variation. **Discussion:** The plate fixation demonstrated greater strength and superior biomechanical tolerance in imperfect reduction scenarios. The main limitation is the lack of fatigue testing and the inability of 3D models to reproduce the structural heterogeneity of human bone. **Conclusions:** Implant selection should be individualized based on fracture stability. 3D models provide a reproducible platform for comparative evaluation of osteosynthesis methods, but future studies should include cyclic loading and biological validation.

## 1. Introduction

Proximal metaphyseal-diaphyseal fractures of the fifth metatarsal were described by Jones and are acute injuries caused by sudden inversion or adduction movements and are most commonly seen in athletes, military personnel, and physically active individuals. These fractures are known for their high risk of delayed union or nonunion due to the limited vascular supply to the metaphyseal-diaphyseal area [1,2,3]. Thus, management of this type of fracture is essential for a rapid return to daily life. Although conservative treatment, which is based on functional or plaster immobilization, can be effective in undisplaced fractures of the fifth metatarsal [4], in certain situations surgical intervention is necessary. Cases in which surgical intervention is required are: highly displaced fractures, comminuted fractures and physically active patients. Therefore, Jones-type fractures often require surgical fixation, especially in active individuals. Current surgical fixation methods involve bicortical screws, Kirschner wires, plates, or modern techniques that do not involve metal implants. More recent studies have compared different methods of osteosynthesis and demonstrated the biomechanical superiority of screw fixation, which provides compression at the fracture site, but have shown limitations in terms of torsional and angular stability [5,6]. Similar to vascular stents, where device geometry such as diameter and length directly influences restenosis rates, implant dimensions in orthopaedic fixation may critically affect biomechanical outcomes [7]. In addition to these limitations, subsequent studies have shown that there are numerous complications that can occur at this level, such as: protrusion of the screw head which can cause local irritation of the adjacent soft tissues, which occurs in approximately 45% of cases, deficient osteosynthesis, pseudarthrosis, delayed consolidation [2]. Also, approximately 20–26% of women and 19% of men have a medullary canal with a thickness of less than 4.5 mm, which can make osteosynthesis with a bicortical screw difficult, in which case fixation with a plate and screws is preferred [8].

Plate fixation has been proposed as a biomechanical solution in cases where there is local osteoporosis, increased comminution, or a medullary canal that does not allow screw fixation. It can also be used in cases of refracture or pseudarthrosis. By applying a plate with screws to the plantar-lateral surface, a better distribution of forces and a superior control of fragment rotation are achieved compared to bicortical fixation [9]. Biomechanical studies have shown that plates have a better resistance to bending stresses compared to bicortical screws [6]. From a clinical point of view, plate fixation has been reported to be successful in both primary cases and in revision or pseudarthrosis situations. Studies conducted on elite athletes have demonstrated a high rate of consolidation, with a relatively short time to return to sports activity [10,11,12]. However, the current literature remains limited, with most studies being retrospective with small sample sizes, lacking comparative analyses with bicortical screws.

Another study proposes a modern, minimally invasive alternative based on high-strength sutures, which eliminates the need for a metallic implant and may reduce the risk of local irritation, but this has not been confirmed by biomechanical or in vivo testing [13].

At the same time, the rapid development of 3D printing technology has allowed the creation of experimental models of the fifth metatarsal, which have the ability to accurately reproduce both the geometry and the mechanical properties of real bone. Studies have shown that 3D printed metatarsal bone models can faithfully reproduce the bone structure and can be mechanically comparable to natural bone and CT-based models [14,15]. Furthermore, research in the field of powder-based 3D printing has highlighted the possibility of reproducing bone microstructure and its integration in tissue engineering, which opens perspectives for increasingly realistic and clinically applicable experimental models [16].

Thus, this study aims to perform tests on the 3D printed fifth metatarsal, testing the force to which the implant bone assembly yields, when applying the forces that are found during the phases of walking, comparing the main osteosynthesis methods, namely bicortical screw or plate with screws. Recently, there are no studies that prove, using 3D printed models, that interfragmentary distance represents an important indicator in the chosen treatment method.

By comparing the two fixation methods, the bicortical screw and the plate with screws, we aimed to optimize the surgical procedures in order to regain physical activities as quickly as possible.

## 2. Materials and Methods

The geometric model of the bone structure used in this study was purchased from Zygote, American Fork, UT 84003, USA, one of the most renowned entities specialized in three-dimensional modeling of the human body. Zygote is internationally recognized for the high accuracy of its 3D representations, obtained by computerized tomography (CT) scanning or magnetic resonance imaging (MRI), depending on the anatomical needs targeted [17]. Based on the sections (slices) resulting from these imaging investigations, the company accurately reconstructs three-dimensional geometric models of the human skeleton and organs, subsequently adapting them for the main computer-aided design (CAD) platforms, such as Creo 10, CATIA V5-6R2024, SolidWorks 2025. The models can also be exported in IGS or STP formats, which facilitates their integration into various software applications. In the context of this endeavor, the three-dimensional model of the right foot was purchased, in STP format, from which the 5th metatarsal was extracted in order to perform a 3D print.

**Figure 1:**
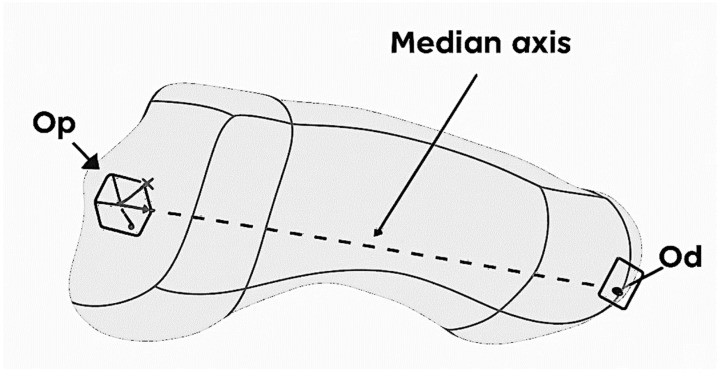
Fifth metatarsal with its geometrical elements.

The STP format was introduced in Catia V5 where the Jones fracture at the level of the 5th metatarsal was defined. The modeling started from defining the median axis of the bone, built between two anatomical landmarks: the proximal point (Op) located in the geometric center of the cubometatarsal joint and the distal point (Od) located in the geometric center of the metatarsophalangeal joint, that can be observed in Figure 1. Reference planes and a local coordinate system Oxyz were generated around this axis, necessary for the parameterized construction.

The Jones fracture was described by three main parameters:

D1 (axial position)—controls the location of the fracture on the median axis of the bone, establishing the level at which the fracture line occurs.

A1 (angular position)—determines the orientation of the fracture around the median axis, allowing the section plane to be rotated at a variable angle.

A2 (fracture line inclination)—defines the angle of inclination of the fracture line in the construction plane, providing flexibility in modeling the propagation direction (e.g., 70°). The fracture line is not strictly straight, but curved, with an amplitude of ±0.5 mm, to faithfully reproduce the clinical appearance.

Based on these parameters and the definition of Jones fracture, the cutting surface was constructed, and by applying the Split command, the metatarsal was divided into two fragments. Subsequently, by using Offset in Assembly mode, the bone fragments were displaced, obtaining a complete simulation of the Jones fracture.

After we have defined the Jones fracture patterns of the fifth metatarsal, bone models were fabricated using multi-material polymer 3D printing, according to previously established parameters, with the aim of obtaining artificial structures with mechanical properties comparable to real bone. The three-dimensional models were made using PolyJet technology using the Stratasys J5 DentaJet printer, which allows the simultaneous deposition of materials with different stiffnesses. The following photopolymer materials were used to reproduce the bone structure: VeroDent™ PureWhite (DEN847) for the rigid components, VeroYellowV (RGD838), VeroCyanV (RGD845) and VeroMagentaV (RGD852) for chromatic control and geometric contrast definition, and MED625FLX™ (Flexible Clear Biocompatible, CL) for the areas with elastic behavior, corresponding to the cancellous segments. The support material used was SUP711™ (Support for Dental Applications), subsequently removed by alkaline solution according to the Stratasys protocol [18]. The models were fabricated from a mixture of rigid and flexible materials, calibrated to approximate cortical and cancellous bone. The values obtained (elastic modulus 1.3–1.6 GPa, compressive strength 65–75 MPa, tensile strength 45–55 MPa) are within the characteristic ratios of human bone tissue, although higher than the ranges reported for metatarsal cortex (elastic modulus 0.38 GPa; compressive strength 40 MPa; tensile strength 50–70 MPa) [19]. Thus, the models proportionally reproduce stiffness and strength, ensuring valid conditions for comparative biomechanical testing, despite the limitation given by the absence of trabecular microarchitecture and anisotropy.

The inclusion criteria were: dimensional deviation ≤±0.3 mm from the CAD model, mechanical parameters within the established ranges and visual integrity without porosities or delaminations. Non-compliant models were excluded.

**Figure 2:**
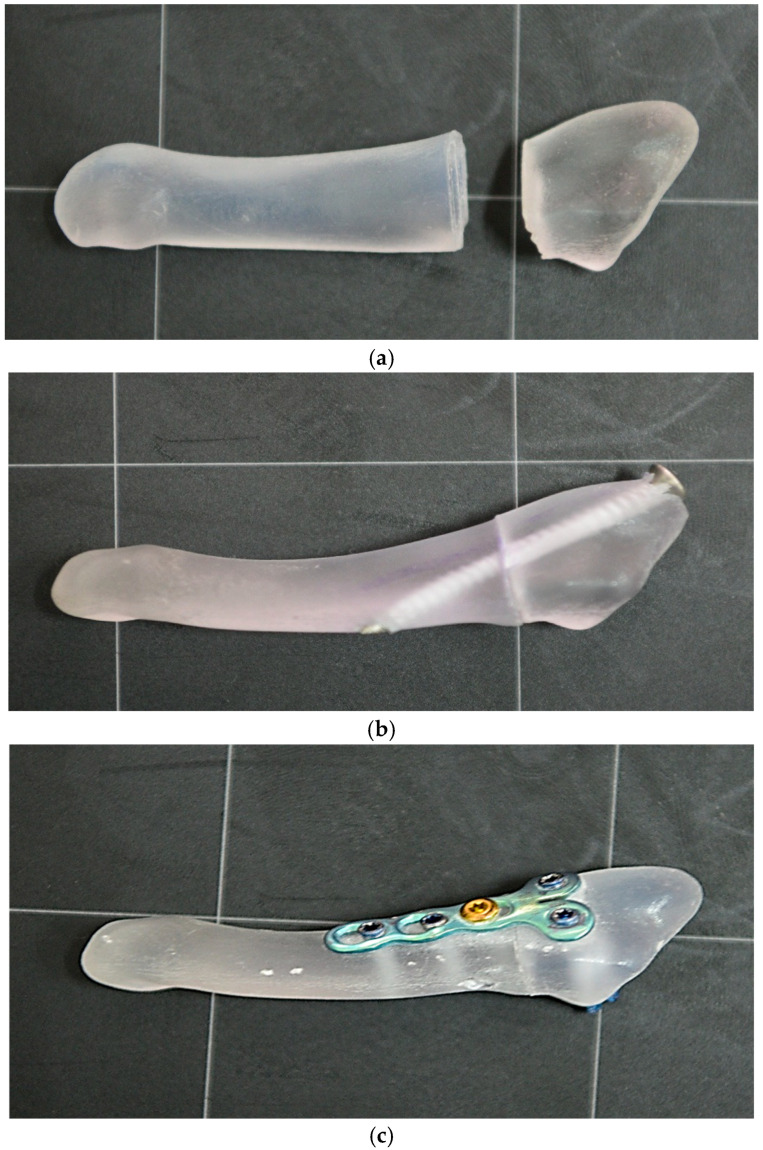
3D printed 5th metatarsal and osteosynthesis methods used, (**a**)—the Jones fracture of the 5th metatarsal printed 3D, (**b**)—fixation of the fracture with a bicortical screw, (**c**)—fixation of the fracture with a plate.

Printing was performed with a 30 μm layer, obtaining a high geometric fidelity, subsequently verified by dimensional measurements, density and structural compliance. The proposed method is economical, reproducible and customizable, confirming the results of other studies that demonstrated the feasibility of 3D printing for biomechanical testing. Recent studies support the use of 3D printing for biomechanical experiments, emphasizing that this method can partially replace both cadaveric samples and standard synthetic models [20,21,22]. An example of the 3D model is presented in Figure 2.

To ensure the accuracy and comparability of the experimental data, potential sources of error were analyzed and minimized. All models were made from the same batch of photopolymer material, using constant printing parameters (layer thickness 30 µm, maximum dimensional deviation ± 0.3 mm). The force sensors were checked and calibrated before the start of the test series, and all assemblies were performed by the same operator. This approach reduced experimental variability and ensured consistency of the results obtained between samples.

A key aspect is the mechanical behavior of these models. Although they cannot fully reproduce the microstructural complexity of human bone, they exhibit more uniform properties than biological samples. Unlike cadaveric bones, which vary significantly in density and elasticity, printed models reduce this heterogeneity, ensuring comparable experimental conditions between samples.

However, it should be emphasized that the lack of trabecular architecture and anisotropic behavior is an important limitation. In biological bone, the heterogeneous mineral distribution, frequently associated with osteoporosis or other metabolic disorders, causes changes in compressive and shear strength. This variability cannot be fully reproduced in printed models. For this reason, data obtained on 3D models should be interpreted as comparative tools, intended to complement and not replace cadaveric studies or in vivo investigations.

To simulate the treatment of Jones fracture of the fifth metatarsal, each model underwent an osteosynthesis procedure. Implants similar to those used in clinical practice were used: a cannulated, partially threaded screw (diameter 4.0 mm, length 40 mm) inserted bicortically and a titanium alloy plate with 5 holes, fixed by 4 locked screws and one non-locked screw. The area corresponding to the fracture focus was left without a screw to avoid influencing the biomechanical results (Figure 2).

**Figure 3:**
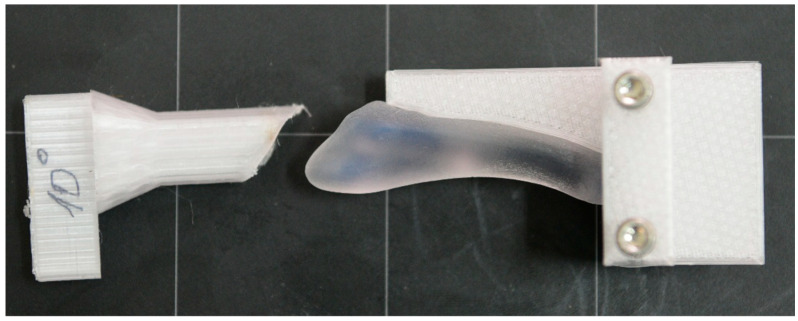
Fixing device for the tests of 3D models.

Subsequently, the osteosynthesized models were subjected to the application of the resultant force of external forces corresponding to the ground reaction (GRF), including the anteroposterior component, vertical component and medio-lateral component, during the phases of walking. The resultant force was reproduced using an experimental device specially designed for this experiment (Figure 3). All mechanical tests were performed using the same axial loading system (Figure 3). For each experimental combination, three distinct specimens (n = 3) were tested, with each model being subjected to a single destructive test, thus ensuring the independence of observations. The order of testing was randomized to eliminate sequence effects.

The applied forces were calibrated according to the values reported in the literature [23,24,25]. Thus, during the heel strike phase, the fifth metatarsal is subjected to the supero-lateral traction of the peroneus brevis tendon (30–40 N) and the infero-medial traction of the plantar fascia (50–70 N). In the intermediate phase, the forces increase significantly, reaching an average axial load of 1.1 N, a lateral traction of approximately 70 N exercised by fascia, and a supero-lateral traction of 400 N exerted by the peroneus brevis.

In the propulsion phase, the resultant force includes an average ground reaction of 35 N, an infero-medial traction of 120 N determined by the windlass mechanism of the fascia, and a lateral traction of approximately 60 N.

**Figure 4:**
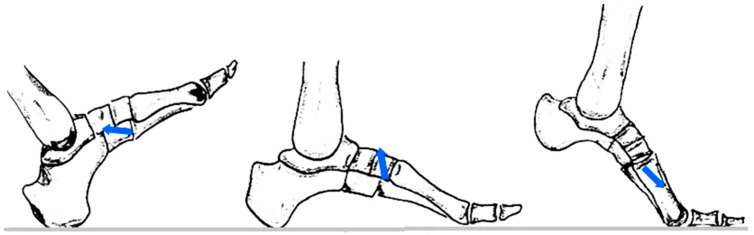
Resultant force applied on fifth metatarsal during the phases of walking.

By calculating the vector resultants, the following values were obtained: in the heel contact phase—85.15 N, oriented at 7° to the bone axis; in the intermediate support phase—452.21 N, at 83°; and in the propulsion phase—122.40 N, oriented at approximately 150°. In the statistical analysis, these values were simplified by correspondences of 0°, 90°, and 180°, to allow factorial evaluation [26]. The obtained resultant is shown in Figure 4.

The yield strength was defined as the maximum force applied before the occurrence of irreversible structural damage to the bone–implant assembly. The criteria adopted to define failure included: (i) the visible initiation of a crack in the model bone or implant, (ii) a sudden and sustained decrease in force (>10% of the maximum value) concomitant with increasing displacement, and (iii) the appearance of a detectable residual plastic deformation after unloading.

An additional objective of the research was to evaluate the influence of the interfragmentary distance on the biomechanical behavior of the fracture. Three experimental scenarios were analyzed: almost perfect reduction (0.1 mm), minimal residual displacement (0.5 mm), and lack of adequate contact (1 mm). The choice of these values was based on clinical observations and data reported in the biomechanical literature, where incomplete reduction of fragments can compromise stability and the healing process [27].

For a systematic investigation, the full factorial experiment (DoE) method was used, simultaneously analyzing the effect of two main factors: the angle of the resultant force applied (α) and the interfragmentary distance (d). Power analysis was performed a priori to determine the minimum sample size required to detect a medium-sized effect (f = 0.25), with a significance level of α = 0.05 and a statistical power of 0.8, resulting in a minimum sample of 24 specimens, with 27 being used in the current study. For each factor, three experimental levels were defined (α = 0°, 90°, 180°; d = 0.1 mm, 0.5 mm, 1 mm), resulting in a total of 27 experimental combinations, each repeated three times for data robustness. The factorial design approach has also been successfully applied in engineering fields, for example in optimizing the power efficiency of RISC processors [28].

This approach allowed not only to identify the direct effects of each parameter on the force at which the implant bone structure yields, but also to evaluate the complex interactions between them. The choice of the factorial methodology is in accordance with current recommendations in applied biomechanics, where the interaction between anatomical factors and functional parameters plays a decisive role in postoperative stability.

The dependent variable investigated was the force at which the assembly formed by the bone fragments and the osteosynthesis system yields, determined in each experimental combination. The complete matrix of the tests, including the coding of the factors, the actual values and the space for recording the results, is presented in Table 1.

The choice of this configuration allowed both the estimation of the pure error and the investigation of the main effects and interactions between factors by means of a replicate ANOVA. The sample size was calculated a priori, based on the detection of a medium intensity effect, with a statistical significance threshold set at α = 0.05.

The two independent factors selected were the angle of the applied force resultant (α) and the interfragmentary distance (d). This choice was based on both clinical observations and data from the literature on the biomechanics of avulsion fractures of the fifth metatarsal.

The defined levels for the α factor (0°, 90°, 180°) were chosen to reflect possible variations in the direction of the force exerted on the bone fragment by the peroneus brevis tendon, the plantar fascia and the ground reaction force (GRF). Musculoskeletal models have shown that as the force vector deviates further from the bone axis, the risk of fragment displacement and osteosynthesis failure increases considerably.

Regarding the d-factor, levels of 0.1 mm, 0.5 mm and 1 mm were established to reproduce clinical bone reduction scenarios: from almost perfect contact between fragments to the existence of significant residual spaces. The increase in the interfragmentary distance is associated with uneven stress distribution and the occurrence of local micromovements, which can delay the healing process and compromise the biomechanical stability of the assembly.

The originality of this study lies in three major aspects: the application of a 3 × 2 replicate factorial design for the simultaneous evaluation of α and d factors, and the integration of a second-order polynomial regression, capable of quantifying the α × d interaction and identifying unstable biomechanical regimes. Through these innovative elements, the research provides practical benchmarks for optimizing fracture reduction and implant orientation in a clinical context.

Statistical analysis was performed using SPSS version 27.0 (IBM Corp., Armonk, NY, USA). A priori power analysis indicated that a minimum of 24 specimens was required to detect a medium effect size (f = 0.25) with a power of 0.80 at α = 0.05. Normality was verified by Shapiro–Wilk testing, and homogeneity of variances was confirmed by Levene’s test. Two-way ANOVA was applied to assess the influence of interfragmentary distance (d) and load angle (α) on maximum failure force (Fmax). Post hoc Tukey’s tests were performed for multiple comparisons. Tukey post hoc analysis revealed significant differences between plate and bicortical screw fixation for all combinations of loading angle (α) and interfragmentary distance (d) (*p* < 0.05), confirming the consistency of the observed effects. For regression analysis, 95% confidence intervals (CI) were calculated for model coefficients, and the adjusted R^2^ was reported to reflect predictive accuracy. Statistical significance was set at *p* < 0.05.

## 3. Results

### 3.1. Analysis of Biomechanical Behavior Using Plate and Screws

To investigate the biomechanical behavior of fifth metatarsal fractures depending on the osteosynthesis method used, a comparative analysis was performed between T-plate fixation and bicortical screw fixation. The specialized literature frequently mentions the use of screws in the treatment of Zones I–II fractures, as they provide bicortical fixation with variable stiffness, influenced by the type of thread, the length of the implant and the degree of compression exerted on the bone fragments [29,30].

Following the experimental research carried out, the values of the force at which the implant fails were obtained, which are presented in Table 2.

Based on the 3 × 2 full factorial design, a second-order polynomial model was constructed to evaluate how the biomechanical factors—interfragmentary distance (d) and force resultant angle (α)—influence the implant failure force in Jones fractures. The model includes linear, quadratic, and interaction terms to capture both main effects and nonlinear and synergistic behaviors.

The resulting mathematical model is presented in Equation (1):

F_max_ = 158.87 − 70.28 × d + 0.141 × α − 10.19⋅d^2^ − 0.0011 × α^2^ (1)

where

F is the maximum force resisted by the osteo-implant assembly, expressed in [N],
d is the distance between bone fragments [mm];
α is the angle of the resultant force [°].

Analysis of variance revealed the following:

The factor d (interfragmentary distance): exerted a significant biomechanical influence on the failure force. Although d was statistically significant (*p* < 0.0001), the quadratic term d^2^ was at the limit of statistical significance (*p* ≈ 0.19). This suggests a nonlinear relationship: after a critical threshold of interfragmentary distance is exceeded, the maximum force supported by the implant decreases rapidly, probably through loss of cortical contact and inefficient load redistribution.

The α factor (the angle of the resultant force): had a direct and significant effect (*p* ≈ 0.0006) on the yield strength. Values close to 90° were associated with notable decreases in implant strength, confirming the critical role of transverse stresses. The quadratic term α^2^ was also significant (*p* < 0.0001), indicating the existence of optimal or suboptimal biomechanical angles for force transmission.

The coefficient of determination (R^2^) of the model was >0.95, confirming that over 95% of the variation in yield strength is explained by the included parameters.

The statistical significance of the model terms was examined through analysis of variance (ANOVA), and the corresponding results are reported in Table 3.

The ANOVA analysis confirms that most of the terms included in the model are statistically significant. This result supports the idea that the implant failure force in the case of Jones fracture does not depend on a single geometric parameter, but on a nonlinear combination between the position of the fragments and the direction of the load vector. The significant relationship of the squared term α^2^ indicates the existence of optimal or critical biomechanical loading zones, with direct relevance for postoperative stability.

Even if the linear influence of the interfragmentary distance (d) is at the limit of statistical significance, the cumulative impact of the terms d^2^ and d × α emphasizes the essential role of correct fracture reduction and precise orientation of the force vector. Thus, implant stability depends not only on the contact of the fragments, but also on the way in which muscle and ground reaction loads are transmitted through the assembly.

The results obtained are consistent with recent studies on the biomechanics of Jones fractures at the level of the fifth metatarsal [31,32], which demonstrated that both the interfragmentary distance and the direction of the applied muscle forces significantly influence the strength of the bone–implant construct. Experimental models integrating the complex interactions between α and d revealed nonlinear mechanical behaviors, confirming the need to use extended regression models to capture these particularities.

Overall, the results obtained contribute to the understanding of the relationship between fracture type, postoperative biomechanical vectors and the stability of the bone–implant construct. The validation of the mathematical model through a coefficient of determination R^2^ = 0.988, correlated with the statistical significance of the main terms (α, d^2^, α^2^), confirms the applicative value of this model both in guiding surgical decisions and in the design of implants or personalized osteosynthesis strategies for Jones fractures of the fifth metatarsal.

Next, the experiments were performed for the T-plate, under conditions identical to those described previously. In this section, the minimum and maximum values of the yield force recorded at the level of the bone–plate assembly in the three phases of gait are presented, highlighting the advantages and limitations of this osteosynthesis method.

**Figure 5:**
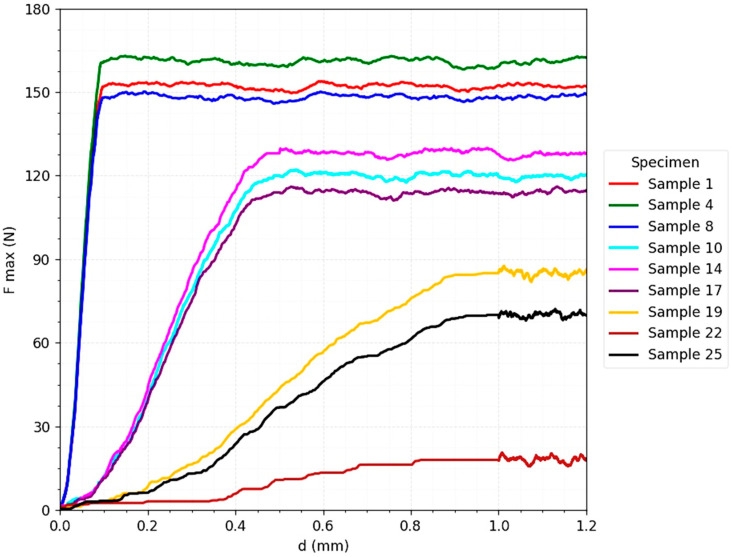
Force–displacement curve for Jones fracture fixation with T-plate (3D printed models).

The Figure 5 for the T-plate strength tests highlights a biomechanical behavior characterized by a rapid increase in the maximum force in the initial phase, followed by reaching a prolonged and relatively constant plateau. This profile indicates a ductile behavior, typical of fixation systems with multiple load distribution. The maximum value of the force supported (F_max) exceeded 150 N under favorable conditions (almost perfect reduction and axial loading), confirming the ability of the plate to maintain the stability of the osteo-implant construct even in scenarios with intense loads.

An important aspect is that, under unfavorable clinical conditions—increased interfragmentation (0.5–1 mm) and oblique loads (90° and 180°)—the resistance level remained significant, ranging between 70–120 N. This result confirms the biomechanical tolerance of the plate and its ability to compensate for the lack of optimal cortical contact or to take up non-uniform loads generated by muscle force vectors and ground reaction [28]. Failure occurred progressively, through gradual deformation of the plate, and not suddenly, which translates into a predictable failure mechanism, associated with a lower clinical risk of instantaneous loss of stability.

### 3.2. Analysis of Biomechanical Behavior Using Bicortical Screw

In continuation of the comparative analysis, additional experiments were performed on the same biomechanical model, replacing the T plate with a bicortical screw. This fixation technique is frequently used in orthopedic practice, especially in Zone I–II fractures of the fifth metatarsal, where the screw provides both intramedullary support and interfragmentary compression. Applied biomechanical studies in the literature have demonstrated that this method offers advantages by reducing micromotions at the fracture site and increasing structural stiffness [29]. The experimental results obtained are presented in Table 4.

Based on the results presented in Table 3, a second-order polynomial regression model was formulated for the bicortical screw fixation system, following the same methodological approach as in the case of plate fixation. By processing the experimental data obtained through a full factorial design of type 3 × 2, with three replicates for each experimental condition, an extended quadratic regression model was established. This model incorporates linear, quadratic, and interaction terms, thereby capturing both the individual and the combined effects of the two investigated factors.

d—interfragmentary distance between bone segments [mm],
α—angle of inclination of the applied resultant force [°].

The mathematical model obtained is presented in Equation (2):

F_max_ = 104.05 − 69.37 × d + 14.69 × d^2^ − 0.114 × α + 0.0001 × α^2^ (2)

where

- F represents the maximum force resisted by the bicortical screw fixation [N];
- d is the distance between the bone fragments [mm];
- α is the angle of the resultant force during walking [°].

From the analysis of the polynomial regression model it results that:

- d (−69.37): the interfragmentary distance has a strong negative influence on the maximum force, increasing the gap decreases fixation strength.
- α (−0.114): the angle of loading has a significant negative effect—greater inclination reduces the force sustained by the assembly.
- d^2^ (+14.69): positive curvilinear effect—at larger distances the decrease tends to stabilize, indicating a nonlinear response.
- α^2^ (+0.0001): the quadratic term of the angle is not statistically significant, suggesting no relevant nonlinear effect of α within the studied range.

The coefficient of determination obtained for this 2nd order polynomial model is R^2^ = 0.987, indicating an excellent predictive capacity of the maximum force.

This means that 98.7% of the variability observed in the experimental data is explained by the proposed model, which validates both the statistical and biomechanical significance of the analyzed facto

The model describes the functional relationship between the interfragmentary distance (d) and the angle of the resultant force (α), highlighting their nonlinear contributions. In particular, the statistical significance of d, d^2^ and α confirms that both the geometry of the fracture and the direction of the load are decisive in determining the fixation strength with bicortical screws.

The ANOVA results corresponding to this model are presented in Table 5, providing statistical and mathematical support for the formulated conclusions.

The analysis of the data presented in Table 5, by applying the ANOVA method to the second-order polynomial model, allowed highlighting the influence of geometric factors on the maximum values of biomechanical stress (σ). The results can be summarized as follows:

The interfragmentary distance d demonstrated a strong statistically significant effect (F = 160.95, *p* < 0.0001), confirming that the separation of bone fragments is the main determinant of the level of biomechanical stress.

The angle α also showed a significant influence (F = 31.50, *p* < 0.0001), which emphasizes the importance of the direction of the force vector in the complex behavior of the bone—implant system.

The quadratic term d^2^ proved significant (F = 9.21, *p* = 0.0063), indicating that the relationship between interfragmentary distance and biomechanical stress is not strictly linear, but presents a biomechanically relevant curvature. This finding suggests the existence of critical distance intervals in which geometric changes generate disproportionate variations in stress.

In contrast, the quadratic term α^2^ did not reach the statistical significance threshold (F = 0.96, *p* = 0.3394), indicating that the relationship between angle and stress remains mainly linear in nature, without highlighting a relevant curvature.

The maximum yield force (Fmax) values for each fixation method are presented as mean ± standard deviation and ranges of variation:

- Plate fixation: 149.78 ± 8.53 N (138–161 N)
- Bicortical screw fixation: 98.56 ± 2.58 N (96–101 N)

These data highlight a clear biomechanical difference between the methods, with an average yield force increase of approximately 45–60% in favor of the plate, under identical conditions of α and d.

In Table 6 there are presented the biomechanical differences between the two osteosynthesis methods.

The results obtained also offer important clinical relevance:

- screw fixation is more suitable for young and active patients, where firm axial compression and high stability of the assembly are sought, in order to prevent secondary displacements.
- plate fixation represents an advantageous solution in the case of unstable fractures, with imperfect reduction, in patients with fragile bone or when it is necessary to limit excessive stresses, offering a more uniform biomechanical behavior in relation to anatomical variabilities.

Thus, the biomechanical model associated with bicortical screw osteosynthesis highlighted an increased sensitivity to α angle variations and a greater rigidity of the system. These particularities may influence the therapeutic choice, depending on the type of fracture and the clinical context of the patient.

Based on these findings, surgical recommendations applicable to the treatment of Jones fractures can be outlined, such as:

- obtaining an optimal reduction to avoid critical interfragmentation distances;
- avoiding implant positioning in situations that generate unstable oblique stresses.

From a biomechanical point of view, the stability obtained by screw fixation depends on its ability to generate a sufficiently high compression to counterbalance the tractions exerted by the plantar fascia and the peroneus brevis tendon. In the vertical support phases, when the stresses are maximum, the lines of force are transmitted predominantly along the bone axis, which determines a beneficial compressive effect at the level of the fracture focus. This mechanism is essential, because maintaining interfragmentary pressure favors the healing process and reduces the risk of secondary instability. The present analysis shows how these loads are distributed on the fifth metatarsal, with emphasis on the fracture area, but also on the diaphysis and distal end of the bone.

**Figure 6:**
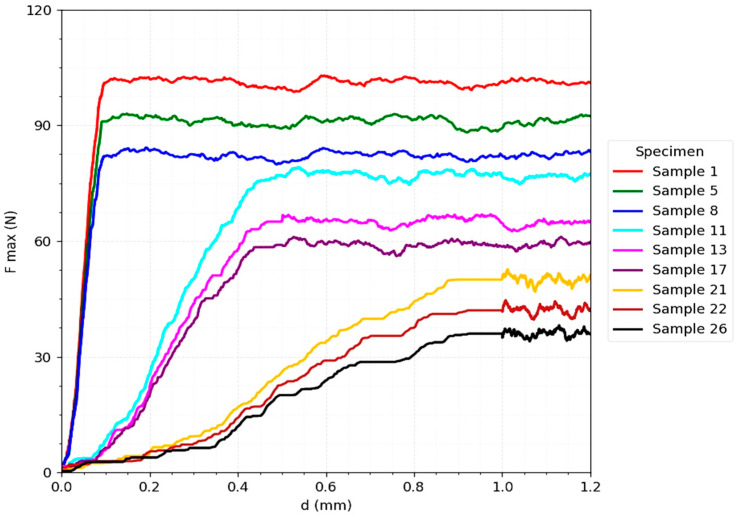
Force–displacement curve for Jones fracture fixation with bicortical screw (3D printed models).

The Figure 6 corresponding to bicortical screw fixation (Figure 2) shows a distinct biomechanical behavior, characterized by a steep slope in the initial phase of the force-displacement curve, indicating a superior stiffness and a good capacity to reduce interfragmentary micromovements immediately after implant placement. However, the maximum values obtained (85–100 N under ideal conditions of reduction and axial loading) are lower than those recorded for the T-plate.

A critical element observed is that, after reaching the maximum resistance threshold, failure occurs suddenly, without a progressive transition zone [33]. This is explained by mechanisms such as thread pullout from the cortex or the occurrence of bone microfractures, which determines a brittle and less predictable behavior. Moreover, the results show a sharp sensitivity to unfavorable clinical factors: at increased interfragmentary distances (0.5–1 mm) or at oblique loads (90° and 180°), F_max drops drastically to 30–50 N, reflecting the vulnerability of the system to geometric variations and load vector deviations [30].

Beyond the raw maximum force values, two additional biomechanical indices were calculated:

(1) the ratio between maximum failure force and estimated body weight (BW, 700 N).
(2) the ductility index, defined as the ratio of the area under the force–displacement curve (AUC) to Fmax.

The ductility index (DI) was calculated as the ratio of the area under the force–displacement curve (AUC) to the maximum force value (Fmax):

(3) $$\mathrm{DI}\;=\;\frac{AUC}{Fmax}$$

This formula provides a quantitative assessment of the plastic behavior of the bone–implant assembly. A higher index reflects a greater progression towards failure and a more likely failure mechanism.

The T-plate resisted up to 0.22–0.25 × BW under unfavorable loading conditions and 0.45 × BW under ideal conditions, whereas the bicortical screw reached only 0.12–0.14 × BW under unfavorable conditions and 0.30 × BW under ideal conditions.

The ductility index was consistently higher for the plate (0.65–0.70) compared with the screw (0.35–0.40), confirming that failure occurred more progressively in the plate system. These indices provide a clinically intuitive interpretation of the mechanical behavior of the constructs.

This lack of biomechanical tolerance limits the applicability of the bicortical screw to well-controlled clinical scenarios—simple fractures, with perfect anatomical reduction and good quality bone.

## 4. Discussion and Limitations

In Jones fractures, the essential parameter describing the postoperative stability of the assembly is not the initial stiffness or the distribution of stresses in the bone, but the maximum force that the bone-implant assembly can withstand before failure. This value defines the real threshold at which the structural integrity of the osteosynthesis is lost and at which the risk of secondary mobilization of the fragment or clinical failure of the treatment appears. In current biomechanical research, the “load to failure” analysis represents the standard by which the performance of an implant can be evaluated under conditions close to the functional reality of walking.

The results obtained in the experimental tests on three-dimensional metatarsal models showed clear differences between the behavior of the T-plate and the bicortical screw. The force-displacement curves related to the bicortical screws revealed a steep initial slope, which confirms the increased stiffness of the assembly immediately after fixation. However, failure occurred suddenly, with a rapid decrease in the value of the force supported once the critical threshold was reached, the mechanism being represented by cortical microfractures and thread pullout. In contrast, the curves corresponding to the plate presented a more moderate slope, but with a wide transition zone to irreversible deformation, which denotes a more ductile behavior and a progressive, much more predictable failure.

The comparative chart of the average values of the force at failure demonstrated that the T-plate withstands loads 20–25% higher than the bicortical screw under conditions of imperfect reduction and at oblique stress angles (α = 90°). This confirms the role of the plate to redistribute the load through multiple anchorage points, ensuring better tolerance in situations where the reduction is not anatomical or when the bone quality is compromised. In contrast, the bicortical screw reached maximum values of the yield force only in scenarios with perfect reduction (interfragmentary ≤ 0.1 mm) and axial loading (α = 0°). Even under these ideal conditions, with the increase of the interfragmentary distance above 0.5 mm or with the oblique orientation of the force, the yield threshold decreased sharply, indicating a marked sensitivity to intraoperative technical variations.

The clinical interpretation of these results is of particular importance. The bicortical screw remains an excellent option in simple, well-reduced fractures and in young patients with high-quality bone. In these situations, the failure threshold is high enough to allow rapid mobilization and early weight-bearing, with a low rate of complications. However, the literature shows that in the case of fractures with difficult reduction or in patients with osteoporosis, failure of screw fixation is more frequent, with rates of refracture and nonunion reaching 15–20% in some series [27].

On the other hand, the T-plate, due to its more tolerant nature, becomes a preferable option when anatomical reduction cannot be achieved, when comminuted fragments are present, or when the bone has low density. The higher failure thresholds in these scenarios confirm the safety of the method in more complicated clinical conditions. Recent studies have shown that patients with plate osteosynthesis had a lower rate of mechanical failure and allowed for earlier progressive loading, even if the initial stiffness of the assembly was lower than in the case of screws.

The relevance of structural fatigue should be discussed especially in the context of high-performance athletes, in whom repeated microstresses can lead to cumulative implant failures. In these cases, the multiple stress distribution offered by plate fixation may represent a significant biomechanical advantage, reducing the risk of fracture associated with single screw fixation.

The clinical discussion must also emphasize the pragmatic aspect of these findings: the choice of implant cannot be uniform for all Jones fractures. The bicortical screw is superior when the surgeon has the guarantee of perfect reduction and good bone quality, conditions in which the high failure threshold provides stability and allows rapid recovery. In contrast, the T-plate becomes the safer solution in cases with a high risk of instability, since the distribution of the load on several anchorage points significantly raises the level of force at which the assembly fails [34,35]. In practical terms, this means reducing the likelihood of reintervention, decreasing the risk of pseudarthrosis and a more predictable functional recovery.

Thus, detailed analysis of the yield strength brings valuable clinical insight and supports the idea of an individualized approach. It is not the initial stiffness or the tradition of the fixation method that should dictate the choice of implant, but the failure threshold under real stress conditions. This approach allows for the optimization of the treatment of Jones fractures and contributes to the reduction of complications and the improvement of long-term functional outcomes.

Although the results obtained are relevant, it is necessary to mention certain limitations of the study:

- absence of in vivo validation: the data come exclusively from tests performed on 3D printed models, which, although they largely reproduce the mechanical characteristics of the bone, do not reflect the trabecular microarchitecture nor the real biological response to the healing process. Validation on cadaveric parts or animal models would be indispensable to confirm the conclusions.
- anisotropy of the material: the structures made using PolyJet technology present uniform mechanical properties, which does not reflect the heterogeneity of human bone tissue. This difference may influence the interaction between the implant and the bone.
- simplification of the loads: the simulated forces were derived from the literature and correspond to the phases of gait, but the individual variability of locomotor biomechanics could not be completely reproduced.
- sample size: the small number of samples tested limits the robustness of the statistical analysis. An extension of the experimental cohorts would allow the evaluation of failure thresholds also in repetitive loading regimes.
- exclusion of soft tissues: the stabilizing or destabilizing effects exerted by periosseous structures were not included in this in vitro model.
- the testing was performed in static mode, without evaluating the behavior under cyclic loading, which may underestimate material fatigue phenomena.
- the small number of samples limits the statistical power of the factorial analysis, and the absence of soft tissues excludes the stabilizing influences of periosseous structures. However, the mechanical uniformity of the printed samples allowed for the reduction of inter-specimen variability, providing a controlled framework for factorial testing.

Another important limitation is that the testing was performed exclusively in static mode, with the determination of the maximum force at failure (ultimate load). Although this method provides precise information about the structural threshold of mechanical failure, it does not capture the fatigue behavior or the effects of repeated micromotions, factors frequently implicated in the delay of Jones fracture healing. The choice of static testing was justified by the objective of the study—the initial comparison of biomechanical performance between fixation systems—with fatigue tests and cyclic numerical modeling to be addressed in future studies.

Although 3D-printed bones cannot fully replicate trabecular anisotropy, our validation of elastic modulus and compressive strength against published cadaveric values supports their relevance as experimental surrogates. By reducing inter-specimen variability, these models provide a controlled environment for factorial testing, which is often difficult to achieve in cadaveric studies. Nevertheless, in vivo confirmation remains essential to determine the clinical transferability of our results.

Another important limitation of the study is that the results were obtained exclusively on 3D printed models, without being validated by tests on cadaveric specimens or in vivo conditions. To strengthen the relevance of these data, it is necessary to extend the research to biomechanical evaluations on cadaveric material, as well as to prospective clinical studies that follow the efficiency of fixation methods, the rate of consolidation and functional recovery [36].

Also, the conduct of controlled clinical trials will allow the investigation of functional recovery, the rate of complications and the results reported directly by patients for each osteosynthesis technique [37]. The integration of this biomechanical, surgical and clinical information is essential for the development of evidence-based therapeutic recommendations applicable to the management of fifth metatarsal avulsion fractures in diverse patient populations.

When compared with previous cadaveric studies, the results of our 3D-printed model tests were consistent in terms of relative implant performance [37] reported that low-profile plates resisted approximately 20% higher torsional loads than intramedullary screws, while [38] demonstrated that screw constructs are more rigid but prone to sudden failure. Our findings align with these results, showing that although bicortical screws achieve greater initial stiffness, their abrupt failure under oblique loading mirrors the brittle behavior reported in cadaveric work.

Our biomechanical findings are consistent with previously reported clinical outcomes. Early intramedullary screw fixation has been shown to reduce time to return to sport compared with casting, but refracture and nonunion rates of up to 15–20% have also been reported [38]. Other authors have emphasized that screws provide excellent stability when anatomical reduction and bone quality are optimal, yet failure remains a concern in elite athletes subjected to repetitive loads [10,31]. In elite athletes, plantar plating has been shown to achieve reliable union with refracture rates below 5%, supporting its use in revision cases and when the medullary canal is narrow [32]. More recent systematic reviews have confirmed that locking plates are associated with lower mechanical failure and earlier progressive loading compared with screws, although screw fixation remains the standard in simple fractures with good reduction [36]. These reports align with our results, highlighting that bicortical screws offer high rigidity but sudden brittle failure, while plates provide a more ductile and predictable profile that translates into greater safety in complex or osteoporotic cases. Taken together, both clinical and biomechanical evidence indicate that implant selection in Jones fractures should be individualized, balancing the technical possibility of achieving anatomical reduction with the patient’s bone quality and functional demands.

Notably, the absolute Fmax values obtained in our study were higher than those reported in cadaveric series, a difference that can be attributed to the homogeneity and increased modulus of the 3D-printed material. This observation reinforces the role of 3D models as reliable comparative tools rather than direct substitutes for biological bone.

The factorial analysis performed in this study also provides practical insights into implant optimization. The significant interaction between fragment displacement (d) and load angle (α) highlights the importance of achieving near-anatomical reduction and of orienting fixation devices to minimize transverse stresses. For implant manufacturers, these results suggest that plate geometry with multiple points of anchorage is more forgiving to reduction errors, while screw fixation requires precise alignment to maintain stability.

Clinically, this translates into a patient-specific approach: screw fixation should be reserved for young individuals with high bone quality and simple fracture lines, whereas plates should be favored in osteoporotic bone, comminuted patterns, or when reduction is suboptimal.

This work brings several original contributions to the biomechanical and surgical literature on Jones fractures:

- Application of a 3 × 3 full factorial design to simultaneously assess the interaction between load angle (α) and interfragmentary distance (d), providing a systematic framework rarely applied in orthopaedic biomechanics.
- Development of second-order polynomial regression models (R^2^ > 0.98), which capture nonlinear behaviors and offer predictive accuracy for implant performance under variable clinical conditions.
- Introduction of a ductility index as an additional biomechanical metric, allowing a clinically intuitive differentiation between brittle (screw) and ductile (plate) fixation mechanisms.
- Use of 3D-printed multi-material bone surrogates validated against cadaveric values, offering an economical and reproducible alternative for preliminary implant testing.

Collectively, these elements strengthen the methodological originality of the study and highlight its relevance not only for implant selection in Jones fractures but also for broader applications in orthopaedic biomechanics.

Regarding external validity, the observed mechanical trends are consistent with published data in cadaveric studies. However, extrapolation of the results to clinical behavior should be done with caution, given the absence of biological healing processes and bone remodeling in 3D printed models. Thus, the conclusions of the present study reflect the comparative mechanical performance of fixation methods, without substituting in vivo clinical evaluation.

Future work should integrate finite element simulations and cadaveric testing to validate these findings and potentially establish individualized guidelines for implant selection in Jones fractures.

## 5. Conclusions

Biomechanical analysis of Jones fractures through the lens of failure force has demonstrated that the real stability of the assembly does not depend exclusively on the initial stiffness of the implant, but on how it resists and behaves until reaching the critical failure threshold.

Bicortical screw osteosynthesis is distinguished by high stiffness and superior capacity to withstand axial forces under ideal conditions, with anatomical reduction and good quality bone. However, its performance collapses with the appearance of minimal reduction errors or oblique stresses, failure being sudden and unpredictable.

In contrast, T-plate fixation presents a greater biomechanical tolerance to the variability of clinical conditions, resisting higher failure forces in unfavorable scenarios. Failure occurs progressively, through plate deformation and not through instantaneous loss of stability, making it a safer option in complex fractures, in osteoporotic bone or when anatomical reduction cannot be achieved.

The comparison of the two methods suggests that there is no universal solution: the bicortical screw is the optimal method for simple and well-reduced fractures, while the T-plate becomes preferable in difficult cases, with a high risk of instability. The choice of implant must therefore be individualized, taking into account the quality of the bone, the accuracy of the reduction and the functional profile of the patient.

From a clinical perspective, these results confirm that the integration of the failure threshold analysis in the biomechanical assessment brings added objectivity and can guide the surgeon in the selection of the safest and most effective osteosynthesis method for Jones fractures.

Future research should validate these in vitro results through cadaveric models and clinical trials, integrating finite element simulations to further optimize implant design and patient-specific fixation strategies reinforcing that surgical decision-making in Jones fractures should be individualized based on fracture morphology, bone quality, and patient demands, rather than guided by a universal protocol.

## Figures and Tables

**Table 1:** Experiment matrix for the full factorial design with two factors (3 levels).

No. Crt.	The Distance Between the Bone Fragments, d (mm)	The Angle of Inclination of the Resultant Force During Walking α (°)	The Maximum Values of the Force, F max (N)	**Sample**
1.	0.1	0		1
2.	0.1	0		2
3.	0.1	0		3
4.	0.1	90		1
5.	0.1	90		2
6.	0.1	90		3
7.	0.1	180		1
8.	0.1	180		2
9.	0.1	180		3
10.	0.5	0		1
11.	0.5	0		2
12.	0.5	0		3
13.	0.5	90		1
14.	0.5	90		2
15.	0.5	90		3
16.	0.5	180		1
17.	0.5	180		2
18.	0.5	180		3
19.	1	0		1
20.	1	0		2
21.	1	0		3
22.	1	90		1
23.	1	90		2
24.	1	90		3
25.	1	180		1
26.	1	180		2
27.	1	180		3

**Table 2:** Force values σ for the full factorial design with two factors (3 levels) and 3 replicates for each level.

No. Crt.	The Distance Between the Bone Fragments, d (mm)	The Angle of Inclination of the Resultant Force During Walking α (°)	The Maximum Values of the Force, F max (N)	**Sample**
1.	0.1	0	152	1
2.	0.1	0	150	2
3.	0.1	0	148	3
4.	0.1	90	161	1
5.	0.1	90	158	2
6.	0.1	90	159	3
7.	0.1	180	140	1
8.	0.1	180	142	2
9.	0.1	180	138	3
10.	0.5	0	120	1
11.	0.5	0	118	2
12.	0.5	0	116	3
13.	0.5	90	125	1
14.	0.5	90	128	2
15.	0.5	90	127	3
16.	0.5	180	112	1
17.	0.5	180	114	2
18.	0.5	180	113	3
19.	1	0	85	1
20.	1	0	82	2
21.	1	0	83	3
22.	1	90	78	1
23.	1	90	76	2
24.	1	90	77	3
25.	1	180	70	1
26.	1	180	68	2
27.	1	180	69	3

**Table 3:** Parameters determined following ANOVA analysis.

Term	F-Value	*p*-Value (PR(>F))	Statistical Significance
**d**	55.87	<0.0001	Highly significant
**α**	17.1	0.0006	Highly significant
**d^2^**	1.79	0.195	Not significant
**α^2^**	33.35	<0.0001	Highly significant
**R^2^**			0.9886

**Table 4:** F values for the full factorial design with two factors (3 levels).

No. Crt.	The Distance Between the Bone Fragments, d (mm)	The Angle of Inclination of the Resultant Force During Walking α (°)	The Maximum Values of the Force, Fmax (N)	**Sample**
1.	0.1	0	101	1
2.	0.1	0	96	2
3.	0.1	0	99	3
4.	0.1	90	88	1
5.	0.1	90	91	2
6.	0.1	90	85	3
7.	0.1	180	79	1
8.	0.1	180	82	2
9.	0.1	180	76	3
10.	0.5	0	73	1
11.	0.5	0	77	2
12.	0.5	0	71	3
13.	0.5	90	65	1
14.	0.5	90	61	2
15.	0.5	90	63	3
16.	0.5	180	56	1
17.	0.5	180	59	2
18.	0.5	180	54	3
19.	1	0	47	1
20.	1	0	45	2
21.	1	0	50	3
22.	1	90	42	1
23.	1	90	39	2
24.	1	90	41	3
25.	1	180	34	1
26.	1	180	36	2
27.	1	180	32	3

**Table 5:** Parameters determined following ANOVA analysis.

Term	F-Value	*p*-Value (PR(>F))	Significance
**d**	2.42	<0.0001	highly significant
**α**	9.76	<0.0001	highly significant
**d^2^**	8.92	0.0063	highly significant
**α^2^**	2.17	0.3394	not significant
**R^2^**			0.9900

**Table 6:** Comparative analysis between the two osteosynthesis methods.

Criteria	T Plate	Bicortical Screw
Yield force F [N]	138–161	96–101
Significance α (ANOVA)	Significant	Significant
Rigidity	Medium	High
Geometric adaptability	Good	Reduced
Sensitivity to α	Low	High
Sensitivity to d	High	High
Biomechanical behavior	Stable, predictable	Rigid, sometimes unstable
Recommendation	Unstable fractures with imperfect reduction	Fractures with precise reduction

## Data Availability

The original contributions presented in this study are included in the article. Further inquiries can be directed to the corresponding author(s).

## References

[1] Dameron T.B. (1975). Fractures and anatomical variations of the proximal portion of the fifth metatarsal. J. Bone Jt. Surg. Am..

[2] Kavanaugh J.H., Brower T.D., Mann R.V. (1978). The Jones fracture revisited. J. Bone Jt. Surg. Am..

[3] Smith J.W., Arnoczky S.P., Hersh A. (1992). The intraosseous blood supply of the fifth metatarsal: Implications for proximal fracture healing. Foot Ankle.

[4] Biz C., Zamperetti M., Gasparella A., Dalmau-Pastor M., Corradin M., de Guttry G., Ruggieri P. (2018). Early radiographic and clinical outcomes of minimally displaced proximal fifth metatarsal fractures: Cast vs functional bandage. Muscles Ligaments Tendons J..

[5] Heineck J., Costa L., Castro M. (2009). Fixation of fifth metatarsal base Jones fractures: A biomechanical study. Arch. Orthop. Trauma Surg..

[6] Huh J., Glisson R.R., Matsumoto T., Easley M.E. (2016). Biomechanical Comparison of Intramedullary Screw Versus Low-Profile Plate Fixation of a Jones Fracture. Foot Ankle Int..

[7] Niță D., Gurzun M., Chiriac L., Cîrstea A.I., Parepa R.I., Barbilian A.G. (2017). Impact of stent diameter and length on in-stent restenosis after bare-metal stent implantation. Rom. Biotechnol. Lett..

[8] Ochenjele G., Ho B., Switaj P.J., Fuchs D., Goyal N., Kadakia A.R. (2015). Radiographic study of the fifth metatarsal for optimal intramedullary screw fixation of Jones fracture. Foot Ankle Int..

[9] Lo Y.C., Tai T.H., Huang Y.M., Chen C.Y. (2024). Intramedullary Screw versus Locking Plate Fixation for Traumatic Displaced Proximal Fifth Metatarsal Fractures: A Systematic Review. J. Clin. Med..

[10] Porter D.A. (2018). Fifth Metatarsal Jones Fractures in the Athlete. Foot Ankle Int..

[11] Bernstein D.T., Mitchell R.J., McCulloch P.C., Harris J.D., Varner K.E. (2018). Treatment of Proximal Fifth Metatarsal Fractures and Refractures with Plantar Plating in Elite Athletes. Foot Ankle Int..

[12] Goodloe J.B., Cregar W.M., Caughman A., Bailey E.P., Barfield W.R., Gross C.E. (2021). Surgical Management of Proximal Fifth Metatarsal Fractures in Elite Athletes: A Systematic Review. Orthop. J. Sports Med..

[13] Sesti F., Berardi A., Oliva F., Masciangelo R., Maffulli N. (2019). Fifth metatarsal tuberosity Jones fractures: A new surgical technique without metal implant. Muscles Ligaments Tendons J..

[14] Coşkun Z., Çelik T., Kişioğlu Y. (2023). Metatarsal bone model production using 3D printing and comparison of material properties with results obtained from CT-based modeling and real bone. Proc. Inst. Mech. Eng. Part H.

[15] Husemoglu R.B., Baysan G., Ertugruloglu P., Tuç Yücel A., Havıtçıoğlu H. (2020). The Mechanical Comparison of Artificial Bone and 3D Printed Bone Segments. J. Med. Innov. Technol..

[16] Brunello G., Sivolella S., Meneghello R., Ferroni L., Gardin C., Piattelli A., Zavan B., Bressan E. (2016). Powder-based 3D printing for bone tissue engineering. Biotechnol. Adv..

[17] https://www.zygote.com/poly-models/3d-human-collections/3d-male-female-anatomy-collection.

[18] https://www.stratasys.com/en/3d-printers/printer-catalog/polyjet/j5-dentajet/.

[19] Danesi V., Cristofolini L., Juszczyk M.M., Erani P., Viceconti M. (2012). Mechanical properties of the human metatarsal bones. J. Mech. Med. Biol..

[20] Metzner F., Neupetsch C., Carabello A., Pietsch M., Wendler T., Drossel W.G. (2022). Biomechanical validation of additively manufactured artificial femoral bones. BMC Biomed. Eng..

[21] Strand K.S., Silvestro E., Naqvi I., Hast M.W. (2024). Elastic properties of 3D printed clavicles are closer to cadaveric bones of elderly donors than commercial synthetic bones. J. Mech. Behav. Biomed. Mater..

[22] Zheng L., Huang X., Li C., Li P., Lin Z., Huang S. (2022). 3D printed trabeculae conditionally reproduce the mechanical properties of the actual trabeculae—A preliminary study. Heliyon.

[23] Chen Y.N., Chang C.W., Li C.T., Chang C.H., Lin C.F. (2015). Finite element analysis of plantar fascia during walking: A quasi-static simulation. Foot Ankle Int..

[24] Jacob H.A. (2001). Forces acting in the forefoot during normal gait--an estimate. Clin. Biomech..

[25] Kaneko F., Edama M., Ikezu M., Matsuzawa K., Hirabayashi R., Kageyama I. (2020). Anatomic Characteristics of Tissues Attached to the Fifth Metatarsal Bone. Orthop. J. Sports Med..

[26] Erdemir A., Hamel A.J., Fauth A.R., Piazza S.J., Sharkey N.A. (2004). Dynamic loading of the plantar aponeurosis in walking. The Journal of bone and joint surgery. Am. Vol..

[27] Perry J., Burnfield J.M. (2010). Gait Analysis: Normal and Pathological Function.

[28] Oltu O., Voiculescu V., Gibson G., Milea L., Barbilian A., Mastorakis N.E., Demiralp M., Mladenov V., Bojkovic Z. (2008). New Approach on Power Efficiency of a RISC Processor, Proceedings of the 8th WSEAS International Conference on Applied Informatics and Communications, Pts I and II, Rhodes, Greece, 20–22 August 2008.

[29] Duplantier N.L., Mitchell R.J., Zambrano S., Stone A.C., Delgado D.A., Lambert B.S., Moreno M.R., Harris J.D., McCulloch P.C., Lintner D.M. (2018). A Biomechanical Comparison of Fifth Metatarsal Jones Fracture Fixation Methods. Am. J. Sports Med..

[30] Bowes J., Buckley R. (2016). Fifth metatarsal fractures and current treatment. World J. Orthop..

[31] Raikin S.M., Slenker N. (2009). The management of Jones fractures in the elite athlete. Foot Ankle Clin..

[32] Vertullo C.J., Glisson R.R., Nunley J.A. (2004). Torsional strains in the fifth metatarsal: Implications for Jones and stress fracture management. Foot Ankle Int..

[33] Rosenberg G.A., Sferra J.J. (2000). Treatment strategies for acute proximal fifth metatarsal fractures. J. Am. Acad. Orthop. Surg..

[34] Hunt K.J., Anderson R.B. (2011). Management of Jones fractures in athletes. J. Am. Acad. Orthop. Surg..

[35] Kerkhoffs G.M., Versteegh V.E., Sierevelt I.N., Kloen P., van Dijk C.N. (2012). Treatment of proximal metatarsal V fractures in athletes and non-athletes. Br. J. Sports Med..

[36] Dobrotă R.D., Barbilian A.G., Sporea C., Ferechide D. (2024). Transforming the Management of Articular Fractures in the Foot: A Critical Examination of Current Methods and Future Directions: A Review. J. Pers. Med..

[37] DeLee J.C., Evans J.P., Julian J. (1983). Stress fractures of the fifth metatarsal. Am. J. Sports Med..

[38] Mologne T.S., Lundeen J.M., Clapper M.F., O’Brien T.J. (2005). Early screw fixation versus casting in Jones fractures. Am. J. Sports Med..

